# Unlocking the surface chemistry of ionic minerals: a high-throughput pipeline for modeling realistic interfaces

**DOI:** 10.1107/S1600576724001286

**Published:** 2024-03-15

**Authors:** Eric Mates-Torres, Albert Rimola

**Affiliations:** a Departament de Química Universitat Autònoma de Barcelona Campus de la UAB Bellaterra Barcelona08193 Spain; Shiv Nadar Institution of Eminence, India

**Keywords:** *PolyCleaver*, Python, mineral surfaces, interfaces

## Abstract

A systematic procedure is introduced for modeling charge-neutral non-polar surfaces of ionic minerals containing polyatomic anions. By integrating distance- and charge-based clustering to identify chemical species within the mineral bulk, our pipeline, *PolyCleaver*, renders a variety of theoretically viable surface terminations. As a demonstrative example, this approach was applied to forsterite (Mg_2_SiO_4_), unveiling a rich interface landscape based on interactions with formaldehyde, a relevant multifaceted molecule, and more particularly in prebiotic chemistry. This high-throughput method, going beyond techniques traditionally applied in the modeling of minerals, offers new insights into the potential catalytic properties of diverse surfaces, enabling a broader exploration of synthetic pathways in complex mineral systems.

## Abstract 2

*PolyCleaver* is an automated Python-based pipeline for modeling ionic mineral interfaces with polyatomic anions, providing new insights into potential catalytic properties and broadening the exploration of synthetic pathways in complex mineral systems.

## 1. Introduction

Minerals have recently attracted significant attention in the materials chemistry and catalysis fields due to their versatility and chemical diversity, which give them a broad range of potential applications. For instance, silicate-based zeolites and clays have shown great promise as heterogeneous catalysts for relevant organic reactions (Liang *et al.*, 2017 ▸; Tu *et al.*, 2019 ▸). Furthermore, minerals are ubiquitous in nature: although silicates are undoubtedly predominant, the Earth’s mantle bears a wide variety of compounds with mineral origin, including oxides, sulfides, carbonates, phosphates and sulfates, among others (Rubin, 1997 ▸). These compounds, which vary widely in their composition, structural complexity and physico­chemical properties, may have even governed the formation of biomolecules that could have led to the emergence of life on early Earth (Miyazaki & Korenaga, 2022 ▸; Li, 2022 ▸). Some efforts have been dedicated to unraveling the interactions between the surfaces of minerals and relevant molecules to rationalize the existence of astrochemical prebiotic synthetic routes (Signorile *et al.*, 2020 ▸; Campisi *et al.*, 2021 ▸) and to reveal the nature of state-of-the-art catalysts for the synthesis of key chemical feedstocks (Chung *et al.*, 2023 ▸). Most studies on refractory minerals as heterogeneous catalysts, however, converge on the key importance of their atomic surface distribution in the adsorption of molecular species and reaction intermediates, driven by the presence of cationic and anionic molecular species (*e.g.* Mg^2+^/Fe^2+^ and [SiO_4_]^4−^, respectively, in most common silicates). Thus, any effort to study these systems computationally is futile without an accurate atomistic representation of the interface between the surface and the adsorbates of interest.

Atomistic modeling of slab surfaces of ionic solids is not straightforward, as the out-of-plane distribution of the ions along the slab model has implications for its energetic features. Ionic surfaces can be one of the three Tasker types: type I, if each plane in the slab displays a net zero dipole perpendicular to the surface; type II, if they display a stacking sequence of charged planes with a net zero dipole in that direction; and type III, where an asymmetric stacking sequence of charged planes leads to the existence of a dipole, thus inducing an electric field (Tasker, 1979 ▸; Watson *et al.*, 1997 ▸). In the latter, the induced field yields a diverging surface energy, tending to infinity in extended periodic structures, potentially influencing the energetics of any chemical process simulated on these systems. In the case of complex minerals like those mentioned above, cleaving them along planes defined by any set of Miller indices yields type II or, more usually, type III slabs, whose dipoles can partly be neutralized through atomic reconstruction of the slab terminations. In all cases, the generated slabs need to match the stoichiometric ratio of the bulk mineral to ensure a correct charge distribution in the unit cell. In addition, given the presence of ionic species usually forming covalently bound clusters in minerals (*i.e.* those in the classes V to IX in the Nickel–Strunz classification, including carbonates, nitrates, borates, sulfates, phosphates and silicates), generating non-polar and charge-neutral surface slab models of these minerals also requires the preservation of all strong covalent bonds along the cleaving plane. These requirements have substantially hampered the number of theoretical studies on these systems, and only a handful of cases shine light on the structural nature of common silicate minerals (Watson *et al.*, 1997 ▸; Bruno *et al.*, 2014 ▸; Geng & Jónsson, 2019 ▸; de Leeuw *et al.*, 2000 ▸) and their interaction with chemically relevant molecules (Campisi *et al.*, 2021 ▸; Rimola *et al.*, 2020 ▸; Navarro-Ruiz *et al.*, 2014 ▸; Zamirri *et al.*, 2017 ▸; King *et al.*, 2010 ▸; Goumans *et al.*, 2007 ▸) as a first step for the study of primordial prebiotic catalysis on astronomical bodies and/or in early Earth environments. Moreover, the structural complexity of even the simplest nesosilicates (such as olivine) hampers the generation of surface slabs based on observation alone, which has mostly limited studies on this mineral to surface modeling and theoretical analysis of only its seven most stable surface terminations. Thus, if the target is to discover novel synthetic routes using Earth-abundant minerals and/or to propose new pathways accounting for the presence of life-bearing biomolecules, a strategy to model complex ionic minerals must be devised, including the multiple terminations co-existing in the polycrystalline states observed in Earth’s mantle and on astronomical bodies (Molster & Kemper, 2005 ▸; Rimola & Bromley, 2021 ▸; Zamirri *et al.*, 2019 ▸). To this aim, in this article, we propose a general pipeline (implemented in a black-box fashion in the *PolyCleaver* tool) for the generation of non-polar and charge-neutral surfaces of ionic minerals with polyatomic anions, and discuss the potential of this newly generated structural diversity for the automatic screening of molecular adsorption on polycrystalline refractory minerals.

## 2. Procedural generation of non-polar and charge-neutral mineral surfaces

**Figure 1:**
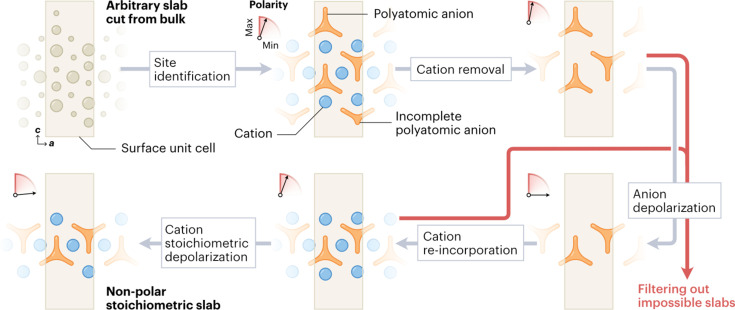
Designing non-polar and charge-neutral mineral surfaces requires an automated pipeline. The surface slab consists of a unit cell (light tan) populated by atomic species (dark tan). An initial estimate of the oxidation states based on the structure stoichiometry followed by clustering methods allows identification of the cationic (blue) and polyatomic anionic (orange) species as a preliminary guess for the slab-generating pipeline (gray arrows). Initial non-polarity is achieved in the anion scaffold after removal of incomplete anionic clusters and cations by sequentially removing full anionic clusters. This is followed by re-incorporation of the cations in their original positions and sequential removal along both ends until global non-polarity and charge neutrality is achieved. Red arrows indicate filtering sub-processes to remove structures with fixed polar patterns.

Cleaving ionic minerals with polyatomic anions in a high-throughput fashion requires providing our baseline chemical structure handling library, *pymatgen* (Ong *et al.*, 2013 ▸), with (i) inference mechanisms to discern the chemical species within the mineral, and (ii) a structural modification strategy to ensure non-polarity and charge neutrality, and to preserve covalently bound molecular scaffolds. In the former, a distance- and charge-based clustering method was used, where the anionic covalent units were defined as the groups of negatively charged atoms surrounding their equally closest positively charged species; all other positive species were deemed cations. This identification is followed by a procedural workflow schematized in Fig. 1 ▸. Given a crystal structure file, our approach works as follows: (i) cleavage of the oxidation state decorated bulk along the desired plane defined by its Miller indices; (ii) removal of any incorrectly cut covalent bonds (revealed by incomplete anionic clusters at the surface or loose negatively charged atoms); (iii) removal of anionic clusters from both sides of the slab to achieve a non-polar anionic substructure; and (iv) generation of an overall non-polar surface structure conditional to a final stoichiometric composition of the overall slab relative to the bulk (see Section 5). Should non-polarity and neutrality not be achievable in any of the ionic substructure, additional filters are put in place that discard the proposed slab model. This process is carried out for a range of non-equivalent terminations to increase the chances of finding a suitable structure. Our pipeline, integrated into the freely available Python package named *PolyCleaver* (see Section 6 for the link to the code), only requires three user inputs, *i.e.* the bulk structure file in CIF format [available in data sets such as the Materials Project (Jain *et al.*, 2013 ▸)], the Miller indices of the desired slabs and their initial thicknesses.

## 3. Assessment of termination-dependent mineral applications

Our pipeline provides a chemically accurate baseline for the analysis of minerals whose predicted activity is mainly termination dependent, *i.e.* where a multifaceted analysis is key to understanding the nature of the mineral–molecule interactions. To exemplify this, we used *PolyCleaver* to model the most stable non-polar and charge-neutral surface terminations, *i.e.* with Miller indices (001), (010), (110), (101), (111), (012) and (102) (Bruno *et al.*, 2014 ▸; de Leeuw *et al.*, 2000 ▸), of the Mg endmember of olivine (forsterite, Mg_2_SiO_4_). Using our approach, *PolyCleaver* automatically generated a range of terminations with various thicknesses. This enabled us to (i) assess the effect that generating a slab by cutting through a given termination plane has on the surface energy, allowing us to pinpoint the most feasible structure, (ii) obtain the optimal slab thickness above which surface energy becomes invariable (as depicted in Fig. S1 in the supporting information), and (iii) build a Wulff construction to reveal the equilibrium shape of the forsterite crystal at 0 K to assess the activity of naturally occurring surface terminations.

**Figure 2:**
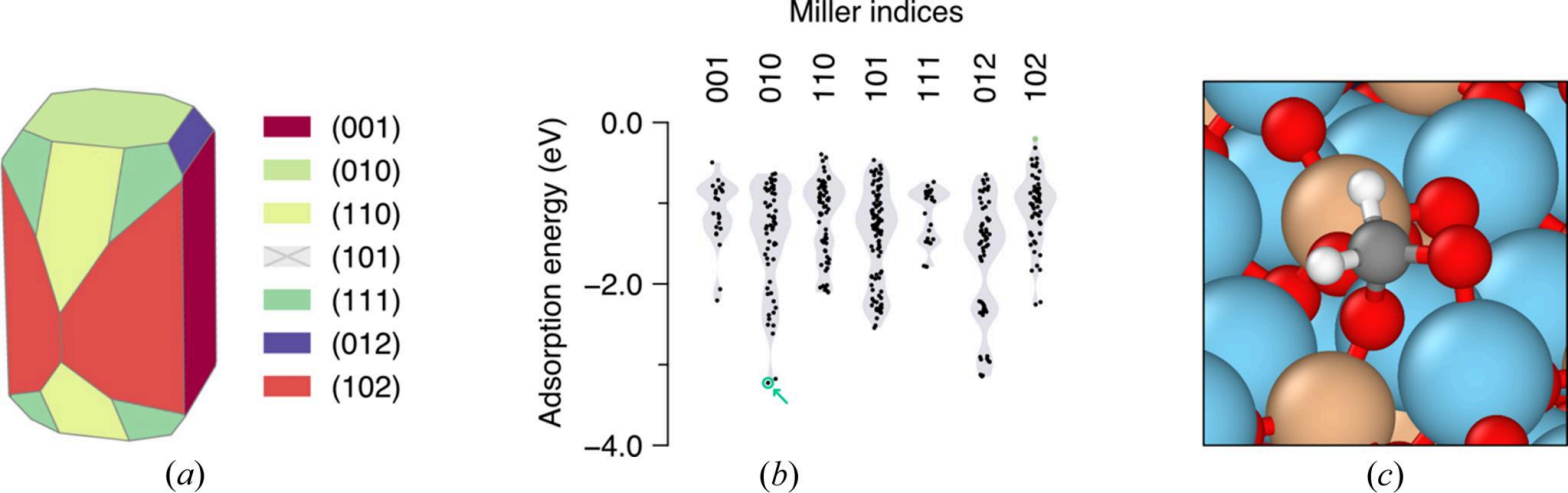
Automatic generation of surfaces allows investigation of accurate crystal morphology and reactivity. (*a*) Equilibrium shape at 0 K of the forsterite crystal built via a Wulff construction from the obtained surface energies. Due to its relatively high surface energy, the (101) surface is not present. (*b*) Adsorption energies of formaldehyde at the semi-empirical quantum mechanical level following an automatic triangulation-based approach on the most common surfaces of formaldehyde. Distinct adsorptions are depicted as dots inside violin plots, which represent the normalized adsorption distribution within each investigated termination. A chemically relevant adsorption point is highlighted in blue and represented in (*c*), corresponding to an activated formaldehyde on the (010) forsterite surface. Atom color code: Mg – blue; Si – tan; C – gray; O – red; H – white.

Surface energies of the studied terminations are reported in Table 1 ▸ and compared with the literature values. Our calculated values are in close agreement with those reported by Watson *et al.* (1997 ▸), de Leeuw *et al.* (2000 ▸) and Bruno *et al.* (2014 ▸). All calculated surface energies are within 6% of at least one value reported in the literature, with one notable exception: the surface energy of our modeled optimal slab for the (101) surface, with an initial thickness of 17.356 Å, is 7.8% lower than the value reported by Watson *et al.*, 17.1% lower than that of Bruno *et al.* and a striking 20.7% lower than that of de Leeuw *et al.* However, in all cases, our pipeline has encountered other less stable surface terminations that display similar energies to the ones reported: an alternative (101) termination with a thickness of 21.695 Å displayed a surface energy of 2.13 J m^−2^, 6.4% and 2.2% lower than the values reported by Watson *et al.* and Bruno *et al.*, respectively. Furthermore, an alternative termination with a starting thickness of 16.123 Å was found to display a surface energy of 1.94 J m^−2^, only 1.1% lower than the value reported by de Leeuw *et al.* Thus, our thorough investigation has revealed that the (101) surface displays a lower surface energy, and thus a higher stability, than the (012) surface, in contrast with previous studies, reinforcing the importance of using an unbiased and automatic approach to find suitable surface terminations. The Wulff-derived equilibrium shape at 0 K arising from the calculated surface energies in this work is represented in Fig. 2 ▸(*a*).

We next sought to assess the ability of all potentially active sites of these surfaces to adsorb formaldehyde (H_2_CO), a multifaceted molecule, also of relevance in prebiotic chemistry as it is a reactant in the formation of sugars and amino acids in the formose reaction and the Strecker synthesis, respectively (Ioppolo *et al.*, 2021 ▸; Aponte *et al.*, 2017 ▸; Meinert *et al.*, 2016 ▸). Due to the geometrical complexity of the generated surfaces, to render the large number of potential adsorption sites, a Delaunay triangulation (Montoya & Persson, 2017 ▸) was used to identify them on the basis of the centers of the outermost exposed atoms of the surface. Fig. S2 illustrates the potential adsorption site grid that our triangulation method yields for the (001) and (123) surface terminations as example cases, showing that the entirety of the unit cell was explored. Then, we took an automatic sequential approach to examine adsorption of the formaldehyde molecule on each identified site from its O atom (which is deemed to be the anchor atom in all instances) until overlap of the ionic radii of O with the atoms on the surface. These preliminary structures were subsequently optimized using semi-empirical methods (see Section 5), providing a broad range of adsorption energies for the modeled surfaces represented in Fig. 2 ▸(*b*). This analysis reveals that, among the most naturally occurring terminations of forsterite, the (010) termination, while being one of the most stable, also appears to be the most reactive towards formaldehyde adsorption and activation, as shown in Fig. 2 ▸(*c*).

**Figure 3:**
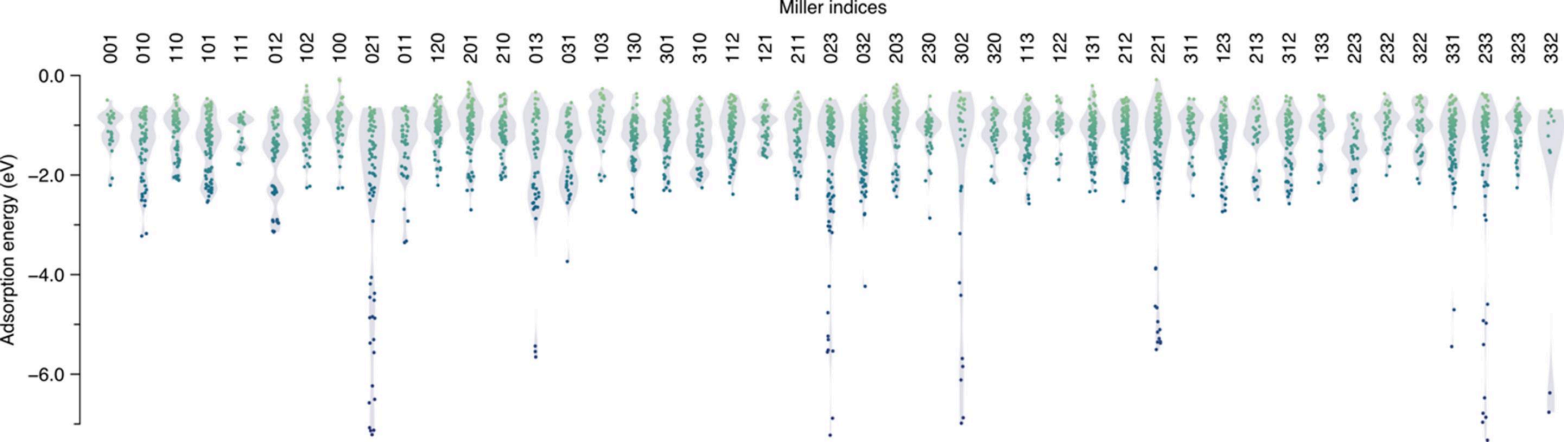
An automatically generated range of surfaces provides a deeper insight into the mineral interfaces. Following an automatic approach based on semi-empirical calculations, the adsorption energies of all distinct adsorption complexes on the 45 different forsterite surface terminations with plane indices from 0 to 3 are depicted as dots. Similarly to Fig. 2 ▸(*b*), violin plots are included to showcase the binding energy distribution within a given plane.

Nonetheless, to showcase the potential of *PolyCleaver* for modeling more complex and novel mineral surfaces, we sought to go beyond literature-abundant surfaces to model all theoretically viable terminations with Miller indices of 0 to 3. Using our pipeline, we were able to quickly obtain a total of 45 surfaces, as shown in Fig. S3, with the intention to model their reactivity towards formaldehyde adsorption. Hence, we used these structures to perform our triangulation-based adsorption analysis yielding a database of 2841 distinct adsorption complexes, whose adsorption energies are depicted in Fig. 3 ▸. This analysis goes far beyond assessing the chemical activity of the most studied forsterite surfaces, highlighting that chemical interest resides not on those but on less stable (Watson *et al.*, 1997 ▸) defect-bearing facets such as (021) or (013), which display some of the strongest molecular adsorptions. Moreover, our calculations show that these strong interactions are associated with a dissociative adsorption of formaldehyde: for instance, the most stable adsorption, corresponding to the lowest point on the (021) surface, corresponds to a doubly dissociative chemisorption of formaldehyde near two highly basic O^2−^ surface sites (as displayed in Fig. S4). These results pave the way for the study of automatically generated surfaces for the catalysis of relevant reaction pathways (*e.g.* the above-mentioned formose reaction and the Strecker synthesis).

## 4. Conclusions

In this article, a straightforward pipeline for the generation of theoretically suitable surfaces of complex ionic minerals (*e.g.* with polyatomic anions) is presented. Our approach, which is implemented in our freely available *PolyCleaver* tool in a black-box fashion, requires only basic user inputs to generate a broad diversity of surface terminations that widely sample the conformational space of the minerals. The ability to generate chemically correct (namely, non-polar and charge-neutral) slab models in a high-throughput fashion is crucial to accurately describe the mineral crystal morphology in a systematic fashion and to accelerate the discovery of novel synthetic routes on the mineral surfaces, as supported by our automated analysis of the interactions between a common pure nesosilicate, forsterite (Mg_2_SiO_4_), and a key prebiotic molecule, formaldehyde. Deployment of this tool will prove useful for modeling other silicate-based systems like cyclo­silicates, inosilicates and phyllosilicates, as well as other potentially polyionic minerals containing covalently bound anionic species such as carbonates, sulfates and phosphates, providing the catalysis and materials science fields with a much-needed diversity of mineral terminations.

## 5. Methods

### 5.1. Surface generation algorithm

To achieve non-polar charge-neutral surface slab models, our pipeline is fed from the tools implemented in the *pymatgen* Python library and adopts a procedural approach. Starting from the mineral bulk structure decorated with the most probable per-atom oxidation states, our algorithm identifies cations and cationic and anionic species of anionic polyhedra (*e.g.* Mg^2+^, Si^4+^ and O^2−^ in Mg_2_SiO_4_, respectively): anion species are deemed those with negative oxidation states, anionic polyhedral centers are classified as those atoms closest to the anions with positive oxidation states, and charge-compensating cations are all positively charged atoms not falling in the previous category. This strategy allows for the analysis of more complex minerals with one or multiple cationic species, such as those with mixed compositions or in the presence of metal dopants. After species classification, our algorithm performs an arbitrary generation of surfaces yielding several Tasker type II or III slabs using the *pymatgen* library (Ong *et al.*, 2013 ▸), considering multiple cuts of a desired thickness and overlooking chemical bonding and overall stoichiometry. Since the non-polar nature of the slab is deemed to be governed by the covalent anionic substructure, a cation-deprived scaffold is used as a basis for polarity correction, by removing undercoordinated anion clusters and sequentially deleting complete clusters at one side of the slab until either non-polarity is achieved or there are no anions left, in which case the slab is discarded. The non-polar anion scaffold is then populated with the original cations, and the structure’s polarity is then corrected in the same fashion while maintaining the anions intact. Finally, cations are sequentially removed from either side of the slab until the stoichiometry of the slab matches that of the bulk. To account for the possibility of multiple corrected Tasker type III slabs converging to the same structure, equivalent slabs are merged in the final data set. This process is automatically carried out for all input planes determined by their Miller indices.

### 5.2. Density functional theory (DFT) calculations

All bulk and surface calculations reported in this work were carried out using the projector-augmented wave (PAW) method (Blöchl, 1994 ▸) and the Perdew–Burke–Ernzerhof (PBE) functional (Perdew *et al.*, 1996 ▸) implemented in the *Vienna Ab-Initio Simulation Package* (*VASP*) code (Kresse & Furthmüller, 1996 ▸). The Mg_2_SiO_4_ bulk, which belongs to the *Pnma* symmetry group and whose structure was obtained from the Materials Project (Jain *et al.*, 2013 ▸), was simulated using a plane-wave energy cut-off of 500 eV. The first Brillouin zone was sampled using a Γ-centered *k*-point grid of 6 × 4 × 2 (resulting in an effective *k*-point density of 20 *k*-points Å), obtained after assessing *k*-point grids from 4 × 2 × 1 up to 18 × 14 × 8 and following a convergence criterion of 1 meV atom^−1^. To obtain the lattice parameters at equilibrium, the energies of the expanded and contracted bulk from 98% to 102% of its original size with a step of 1% were plotted using a Birch–Murnaghan equation of state, yielding a set of lattice parameters of *a* = 4.780, *b* = 6.019 and *c* = 10.273 Å. All automatically generated slabs were optimized with the same parameters by sampling the reciprocal space using the same *k*-point density while only considering the Γ point in the direction perpendicular to the plane. In all cases, all atoms in the structure are allowed to relax freely, thus considering all models to be those of a free-standing slab. Slab surface energies (γ) reported in this work are defined as follows:
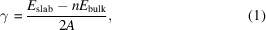
where 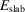 is the energy of the optimized non-polar symmetric slab, 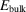 is the energy of the forsterite bulk, *A* is the surface area and *n* is the stoichiometric coefficient of the slab with respect to the bulk. Surface energies were obtained after convergence with respect to the slab thickness, with a convergence criterion of 1 meV atom^−1^. Wulff construction representations were carried out using the *pymatgen* library (Ong *et al.*, 2013 ▸).

### 5.3. Automation of molecular adsorptions

Given the complex nature of the surface, where the vertical position of surface atoms can vary by up to 2 Å on certain terminations, the outermost atoms of the surfaces (*i.e.* those exposed to the adsorbates) were deemed those that passed the condition that no other atoms lie above them within a cylinder of a radius given by the tabulated atomic radius of the target atom. Potential adsorption sites were identified by means of a Delaunay triangulation (Montoya & Persson, 2017 ▸) from the centers of the surface atoms, where top, bridge and face-centered cubic/hexagonal closest-packed (f.c.c./h.c.p.) sites were determined to be the vertices, the edge mid-points and the centers of the triangles, respectively. The formaldehyde adsorbates were aligned towards the surface adsorption sites given an anchor atom (in this case, O) and sequentially approached the surface until contact (*i.e.* until overlap of the ionic radii of the atoms of formaldehyde with those on the forsterite surface). All preliminary structures of the adsorbates on top of the generated terminations of forsterite were optimized using the semi-empirical GFN-xTB method (Grimme *et al.*, 2017 ▸) with periodic boundary conditions using the *tblite* package interfaced with the *Atomic Simulation Environment* (*ASE*) package (Hjorth Larsen *et al.*, 2017 ▸), for the sake of efficiency. In these preliminary calculations, since minimal surface reconfiguration after adsorbate inclusion occurs, atoms belonging to the surface model were kept frozen at their DFT-optimized positions, while atoms of the formaldehyde molecule were allowed to relax freely. The adsorption energy (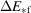) of formaldehyde on the forsterite surfaces at the GFN-xTB level was calculated as
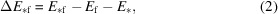
where 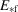 is the GFN-xTB energy of the forsterite–formaldehyde complex, 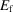 is the GFN-xTB energy of the formaldehyde molecule in the gas phase and 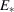 is the GFN-xTB energy of the bare surface.

## 6. Data availability

The authors declare that all data supporting this study are available within the main text. The structures of all the generated surfaces of Mg_2_SiO_4_ are displayed in Fig. S1. All computational data on the surfaces at the DFT level reported in this work, including geometries and energies, and the trajectories of all formaldehyde adsorptions on the generated surfaces of Mg_2_SiO_4_ at a semi-empirical level can be accessed via an ioChem-BD data set (https://doi.org/10.19061/iochem-bd-6-289). The source code of *PolyCleaver* is shared with a GNU General Public License v3.0 and is accessible in the following GitHub repository: https://github.com/ericmates/PolyCleaver. The algorithm for the automatic identification of surface adsorption sites and subsequent approach and optimization of the adsorbates on the surface is available upon request.

## Supplementary Material

Extended data Figs S1-S4. DOI: 10.1107/S1600576724001286/ui5001sup1.pdf

Computational data on the surfaces at the DFT level reported in this work, including geometries and energies, and the trajectories of all formaldehyde adsorptions on the generated surfaces of forsterite at a semi-empirical level.: https://doi.org/10.19061/iochem-bd-6-289

## Figures and Tables

**Table 1:** Surface energies (γ) at 0 K (in J m^−2^) of the predominant terminations of forsterite calculated here and in previous studies Values in parentheses represent the deviation (in %) of our values with respect to those in the literature.

Miller indices (*Pbnm*)	Calculated γ	Watson *et al.* (1997 ▸)	De Leeuw *et al.* (2000 ▸)	Bruno *et al.* (2014 ▸)
(010)	1.71	1.61 (+6.4%)	1.74 (−1.6%)	1.78 (−3.8%)
(111)	1.76	1.80 (−2.4%)	1.81 (−2.9%)	1.84 (−4.5%)
(001)	1.32	1.28 (+3.3%)	1.28 (+3.3%)	1.22 (+8.4%)
(101)	1.81	2.28 (−20.7%)	1.96 (−7.8%)	2.18 (−17.1%)
(110)	1.68	1.81 (−7.3%)	1.88 (−10.7%)	1.78 (−5.7%)
(102)	1.41	1.56 (−10.0%)	Not reported	1.36 (+3.3%)
(012)	1.92	1.95 (−1.5%)	1.94 (−1.0%)	1.90 (+1.1%)
