# Supplementary material for: Unlocking the surface chemistry of ionic minerals: a high-throughput pipeline for modeling realistic interfaces
Source: J Appl Crystallogr. 2024 Mar 15;57(Pt 2):503–8. doi: 10.1107/S1600576724001286 (PMC11001413; doi:10.1107/S1600576724001286)
Supplement: Supplementary file 1 [file j-57-00503-sup1.pdf]

JOURNAL OF APPLIED CRYSTALLOGRAPHY

Volume 57 (2024)

Supporting information for article:

Unlocking the Surface Chemistry of Ionic Minerals: a High-Throughput Pipeline for Modeling Realistic Interfaces

Eric Mates-Torres and Albert Rimola

Extended Data

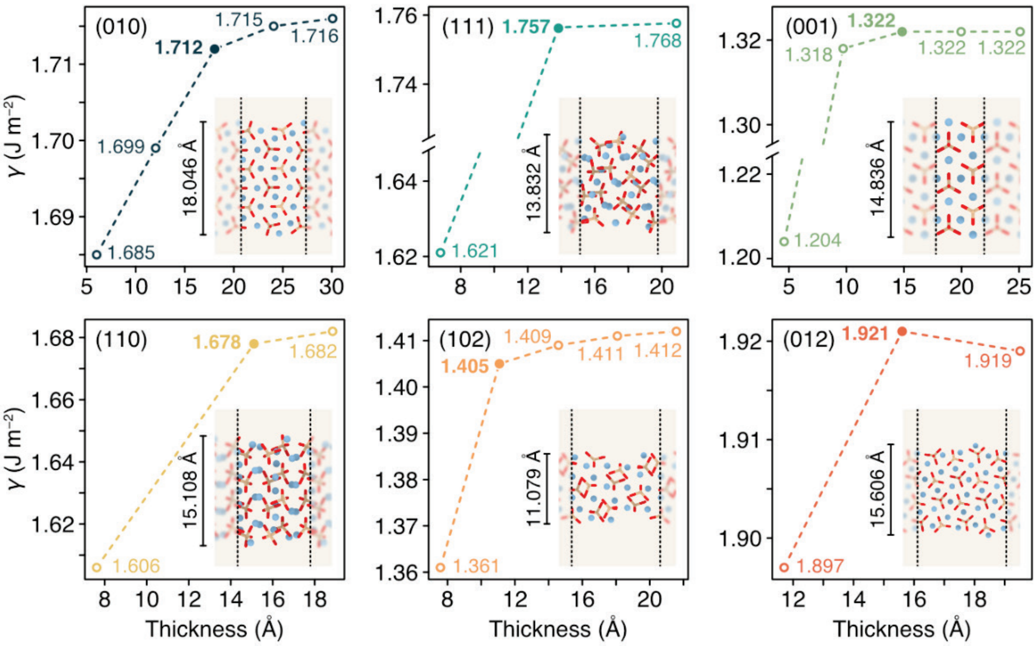

Figure S1 **Extended Data Fig. 1 | Surface energy of the slabs converges at medium slab thickesses.** Each plot depicts the surface energy (in J m^{-2}) with respect to the modelled slab thickness for the six most stable surfaces: (010), (111), (001), (110), (102) and (012). Points corresponding to the surface energy with the converged thickness are depicted as filled dots. Note that, due to the large number of potential terminations of the (101) surface, giving rise to a wide variety of surface energies, convergence tests for this surface were not conducted; instead, its surface thickness was determined to be above 15 Å, in line with the convergence results presented herein. Thus, the panel for the (101) surface is omitted. All plots contain an insert with a side view representation of the slab model with converged slab thickness, which is displayed on its left. Surface unit cells are denoted with a dotted line. Mg^{2+} cations are depicted as blue spheres, while SiO_4^{4-} polyatomic anions are depicted as yellow and red tetrahedra (denoting Si and O atoms, respectively).

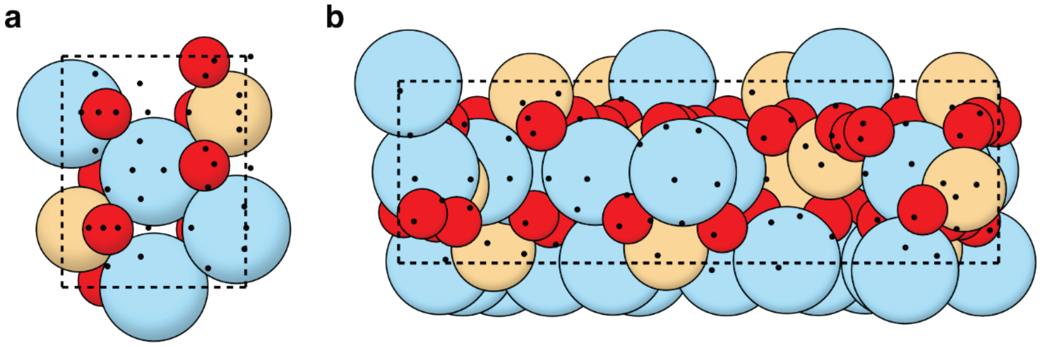

Figure S2 **Extended Data Fig. 2 | Initial adsorption site grid generated by triangulation of surface atomic centers.** Top views of the unit cells of two structurally distant cases are depicted: a) a simple (001) surface, and b) a more complex (123) surface. Coordinates of the identified adsorption sites are represented as black dots, while the unit cell is depicted as a black dashed line. Mg, Si and O atoms are depicted in blue, tan and red, respectively.

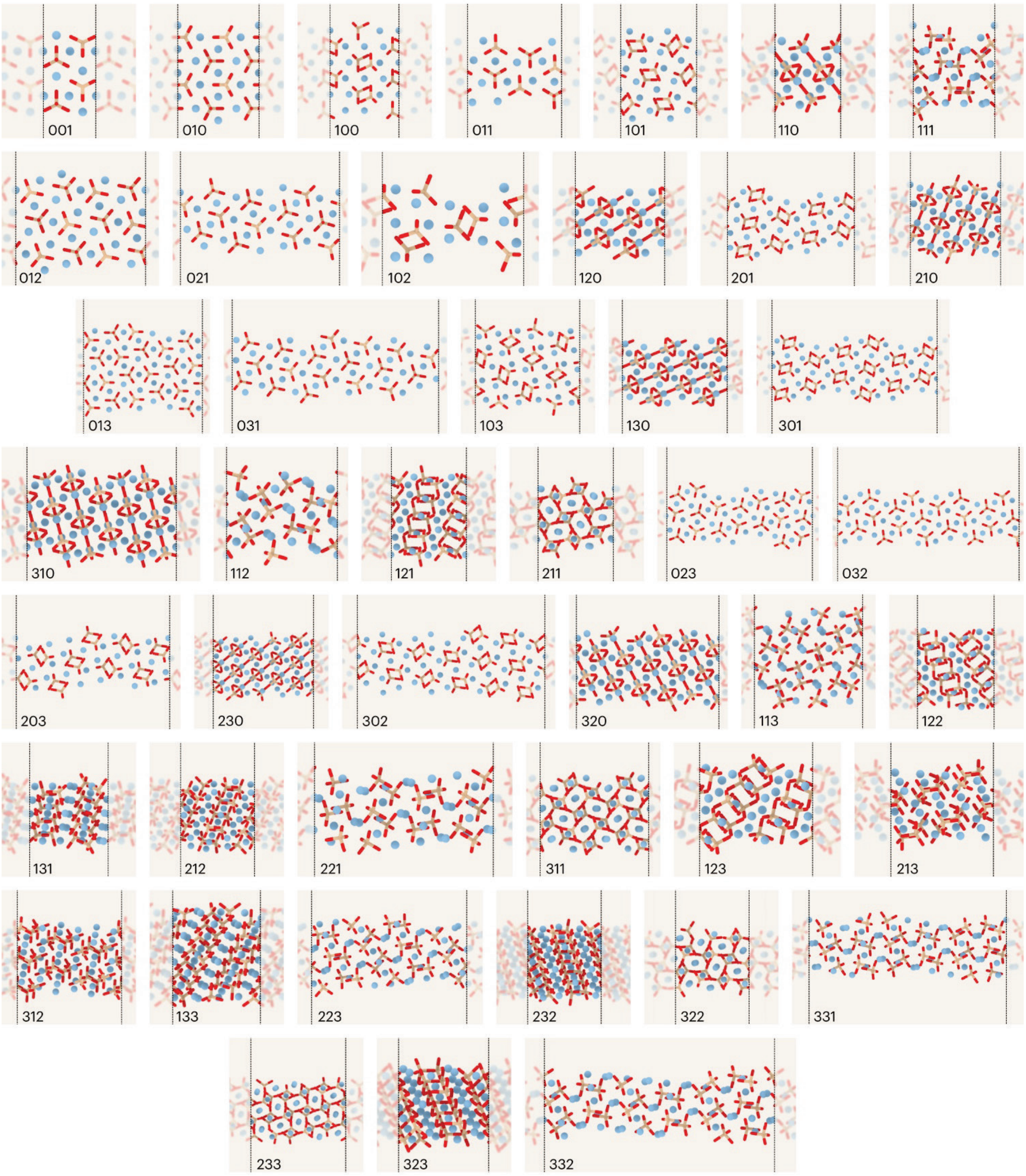

Figure S3 Extended Data Fig. 3 | Slab models of the simulated surfaces for the Mg-end member of the olivine silicate. All structures are depicted with a p(1×1) periodicity, their representations oriented along the a axis. The Miller indices corresponding to the modelled planes are depicted at the bottom of each slab representation. Surface unit cells are denoted with a dotted line. Mg^{2+} cations are depicted as blue spheres, while SiO_4^{4-} polyatomic anions are depicted as yellow and red tetrahedra (denoting Si and O atoms, respectively).

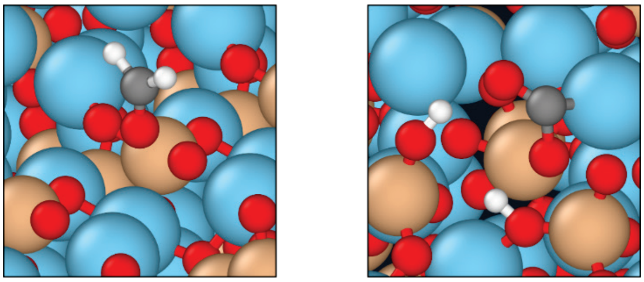

Figure S4 **Extended Data Fig. 4 | Defect-bearing terminations allow complex chemistry.** In the panels, an isometric representation of the adsorption, activation and dehydrogenation of formaldehyde on its most exothermic configuration on the (021) surface of forsterite is depicted, both in its initial (left) and final conformation (right). Atom color code: Mg – blue; Si – tan; C – gray; O – red; H – white.
